# Neutrophilic Asthma Is Associated With Smoking, High Numbers of IRF5+, and Low Numbers of IL10+ Macrophages

**DOI:** 10.3389/falgy.2021.676930

**Published:** 2021-06-21

**Authors:** Nil Turan, T. Anienke van der Veen, Christina Draijer, Fatemeh Fattahi, Nick H. ten Hacken, Wim Timens, Antoon J. van Oosterhout, Maarten van den Berge, Barbro N. Melgert

**Affiliations:** ^1^GlaxoSmithKline, Allergic Inflammation Discovery Performance Unit, Respiratory Therapy Area, Stevenage, United Kingdom; ^2^Department of Molecular Pharmacology, Groningen Research Institute for Pharmacy, University of Groningen, Groningen, Netherlands; ^3^University Medical Center Groningen, Groningen Research Institute for Asthma and Chronic Obstructive Pulmonary Disease (COPD), University of Groningen, Groningen, Netherlands; ^4^Department of Pulmonology, University Medical Center Groningen, University of Groningen, Groningen, Netherlands; ^5^Department of Pathology and Medical Biology, University Medical Center Groningen, University of Groningen, Groningen, Netherlands

**Keywords:** biopsy, inflammatory endotypes, macrophages subtypes, FEV1, CD206, M1, M2

## Abstract

Asthma is a heterogenous disease with different inflammatory subgroups that differ in disease severity. This disease variation is hampering treatment and development of new treatment strategies. Macrophages may contribute to asthma phenotypes by their ability to activate in different ways, i.e., T helper cell 1 (Th1)-associated, Th2-associated, or anti-inflammatory activation. It is currently unknown if these different types of activation correspond with specific inflammatory subgroups of asthma. We hypothesized that eosinophilic asthma would be characterized by having Th2-associated macrophages, whereas neutrophilic asthma would have Th1-associated macrophages and both having few anti-inflammatory macrophages. We quantified macrophage subsets in bronchial biopsies of asthma patients using interferon regulatory factor 5 (IRF5)/CD68 for Th1-associated macrophages, CD206/CD68 for Th2-associated macrophages and interleukin 10 (IL10)/CD68 for anti-inflammatory macrophages. Macrophage subset percentages were investigated in subgroups of asthma as defined by unsupervised clustering using neutrophil/eosinophil counts in sputum and tissue and forced expiratory volume in 1 s (FEV1). Asthma patients clustered into four subgroups: mixed-eosinophilic/neutrophilic, paucigranulocytic, neutrophilic with normal FEV1, and neutrophilic with low FEV1, the latter group consisting mainly of smokers. No differences were found for CD206+ macrophages within asthma subgroups. In contrast, IRF5+ macrophages were significantly higher and IL10+ macrophages lower in neutrophilic asthmatics with low FEV1 as compared to those with neutrophilic asthma and normal FEV1 or mixed-eosinophilic asthma. This study shows that neutrophilic asthma with low FEV1 is associated with high numbers of IRF5+, and low numbers of IL10+ macrophages, which may be the result of combined effects of smoking and having asthma.

## Introduction

Asthma is a chronic respiratory disease characterized by airway inflammation and heterogeneity in disease pathogenesis and severity, symptom triggers, and treatment responses. Cohort studies like UBIOPRED and SARP using clinical and transcriptomics data have shown the existence of different inflammatory subgroups (phenotypes) within asthma that are likely driven by different mechanistic pathways (endotypes) (1–3). This variation within the disease has hampered current treatment of patients and the development of new treatment strategies (4). A better understanding of the underlying disease mechanisms within the different phenotypes is imperative to improve treatment.

Macrophages are among the most abundant immune cells within the lung and they can contribute to development and progression of asthma as well as prevent asthma development in mouse models through different functional subtypes associated with Th1/Th17-driven inflammation, Th2-driven inflammation, or resolution of inflammation (5–21). We have previously shown that asthma patients have both more interferon-regulatory factor 5 (IRF5)-positive macrophages, associated with Th1/Th17 inflammation, and CD206-positive macrophages, associated with Th2 inflammation, while having fewer anti-inflammatory interleukin-10 (IL10)-positive macrophages as compared to healthy controls (6, 9). We also showed that these IRF5+ macrophages associate with having worse and IL10+ macrophages with having better lung function. However, no studies as of yet have addressed whether the different phenotypes of asthma associate with the presence of a specific macrophage subtype, while this may give more insight into the underlying disease mechanisms of each of these asthma phenotypes. We therefore investigated different macrophage subtypes in relation to asthma phenotypes in a well-characterized asthma cohort. We used several parameters like neutrophil and eosinophil counts in tissue and sputum and lung function to cluster patients into inflammatory subgroups in an unbiased way. As eosinophilic asthma is generally associated with Th2-high inflammation and neutrophilic asthma with Th1/Th17 inflammation (3, 22), we hypothesized that eosinophilic asthma would be characterized by having Th2-associated CD206+ macrophages, whereas neutrophilic asthma would have Th1/Th17-associated IRF5+ macrophages.

## Materials and Methods

### Subjects

Bronchial wall biopsies of asthma patients (*n* = 138) originated from cohorts previously studied by our group (6, 23–25). The experimental protocols were approved by the Medical Ethical Committee of the UMCG (METc 2007/007, clinicaltrials.gov NCT00848406) and all subjects gave written informed consent. We used eosinophil and neutrophil cell counts from biopsies and sputum samples and pre-and post-bronchodilator lung function to cluster asthma patients into inflammatory endotypes. As we did not have all data for all patients, we finally included 79 patients for the cluster analysis. Table 1 presents the clinical characteristics of the 79 asthma patients. For comparison, we included data for 50 healthy subjects that we also studied before (6).

**Table 1:** Characteristics of patients in the different asthma subgroups and healthy controls (added for comparison).

**Patient characteristics**	**Healthy (*n* = 50)**	**Mixed eosinophilic (*n* = 14)**	**Neutrophilic low FEV_**1**_ (*n* = 24)**	**Neutrophilic normal FEV_**1**_ (*n* = 21)**	**Paucigra-nulocytic (*n* = 20)**	***p-*value (without healthy)**
Age (years)	46 ± 18	49 ± 11	54 ± 9	47 ± 12	47 ± 13	0.08
Gender (female/male)	21/29	6/8	7/17	13/8	10/10	0.17
BMI	24.2 ± 3.9	26.6 ± 5.2	25.8 ± 4.5	29.0 ± 5.9	27.9 ± 4.4	0.19
Atopy (yes/no/unknown)	0/50/0	12/2/0	18/5/1	14/7/0	12/5/3	0.58
ICS use (yes/no)	0/50	7/7	11/13	10/11	15/5	0.20
Smoking (pack years)	5.5 ± 11	0.3 ± 0.7	16 ± 14	6 ± 10	6 ± 10	**1.4E-04**
Current smokers (yes/no)	17/33	0/14	10/14	1/20	4/16	**2.4E-03**
**Lung function parameters**						
FEV_1_ (% predicted)	103 ± 13	101 ± 18	73 ± 13	101 ± 8	101 ± 13	**1.1E-07**
FEV_1_ after β_2_-agonist (% predicted)	106 ± 13	101 ± 18	82 ± 13	107 ± 8	101 ± 13	**2.1E-08**
FEV_1_/FVC	78 ± 6	68 ± 11	58 ± 10	78 ± 7	70 ± 10	**2.2E-07**
MEF_50_ (% predicted)	NA	70 ± 30	45 ± 16	83 ± 23	74 ± 26	**4.8E-06**
PC_20_ AMP (mg/mL)	621 ± 96	154 ± 266	160 ± 229	482 ± 260	249 ± 298	**3.5E-04**
**Biopsy cell counts**						
Eosinophils (number per 0.1 mm^2^ tissue area)	1.8 ± 3.6	18.9 ± 8.8	1.7 ± 2.5	2.6 ± 3.3	2.3 ± 2.1	**6.8E-19**
Eosinophils (positive pixels per 0.1 mm^2^ tissue area)	NA	2007 ± 1650	163 ± 155	235 ± 405	269 ± 329	**1.9E-10**
Neutrophils (number per 0.1 mm^2^ tissue area)	NA	6.6 ± 5.1	7.8 ± 6.2	11.4 ± 6.7	2.3 ± 2.1	**1.7E-05**
**Sputum cell counts**						
Neutrophils (%)	49 ± 22	49 ± 19	66 ± 18	58 ± 24	40 ± 10	**1.0E-04**
Eosinophils (%)	0.5 ± 0.9	10.2 ± 17.4	1.4 ± 1.6	0.5 ± 0.7	1.5 ± 2.3	**1.5E-03**

*The p-value is derived from a one-way ANOVA comparing differences between the asthma subgroups excluding the healthy individuals for continuous data and a Chi-square-test for nominal data. Data are presented as mean ± standard deviation. NA: not assessed. Bold values indicate significant differences*.

### Asthma Patients

Smoking and non-smoking patients with asthma, aged 19–71 years, either using or not using inhaled corticosteroids (ICS), were recruited from research cohorts in Groningen, the Netherlands. They had a doctor's diagnosis of asthma according to GINA guidelines and documented reversibility and bronchial hyperresponsiveness (BHR) to histamine in the past. Asthma patients were extensively re-examined [see for study design references (6, 23–25)] and were included in the study if they showed a positive AMP provocation test (PC_20_AMP < 320 mg/mL), or if this was >320 mg/mL a positive histamine provocation test (PC_20_histamine < 32 mg/mL). Non-smokers were defined as subjects who had not smoked during the last year, had never smoked for as long as 1 year, and had not smoked more than 0.5 pack years. The main exclusion criteria were: FEV1 < 1.2 L, chronic obstructive pulmonary disease, bronchiectasis, upper respiratory tract infection (e.g., colds) and/or use of anti- biotics or oral corticosteroids within 2 months prior to inclusion in the study.

### Healthy Individuals

Healthy individuals were included if they met the following criteria: (1) normal pulmonary health according to the physician, (2) normal spirometry defined as FEV_1_ ≥ 80% predicted, an FEV_1_/forced vital capacity (FVC) greater than the lower limit of normal, (3) no bronchodilator reversibility defined as an increase in FEV_1_ < 10% of the predicted value after administration of 400 μg salbutamol, and (4) no bronchial hyperresponsiveness to methacholine. Non-smokers were defined as subjects who had not smoked during the last year, had never smoked for as long as 1 year, and had not smoked more than 0.5 pack years.

### Lung Function

A daily-calibrated spirometer was used to perform lung function tests according to standardized guidelines as previously described (26). FEV_1_ was measured with a calibrated water-sealed spirometer (Lode Spirograph D53, Lode Instruments, Groningen, The Netherlands) according to standardized guidelines (27). After administration of 400 μg albuterol the reversibility of FEV_1_ (% predicted) was measured. Provocation tests were performed as published previously (6, 23–25). Subjects received an initial nebulized 0.9% saline challenge, followed by doubling concentrations of AMP (0.04–320 mg/mL) by 2-min tidal breathing at intervals of 5 min. BHR to histamine was measured by doubling concentrations ranging from 0.13 to 32 mg/mL using the 30-s tidal breathing method (28).

### Sputum Induction and Processing

Sputum was induced by inhalation of 5% hypertonic saline aerosols over three consecutive periods of 5 min. Processed whole sputum samples were stained with May Grünwald Giemsa to obtain cell differentials by counting total 600 viable, non-squamous cells. Sputum was not used if the percentage of squamous cells was >80% or if the total number of non-squamous cells was <600.

### Collection and Processing of Bronchial Biopsies

Bronchial biopsies were collected with a flexible bronchoscope (type Olympus BF P20 or BF XT20; Olympus, Center Valley, PA) under local anesthesia. The biopsies were obtained from segmental divisions of the main bronchi and thereafter fixed in 4% formalin. After processing, the biopsies were embedded in paraffin and cut into sections of 3 μm thickness.

### Histology

All immunohistochemical stainings (except for macrophages) were performed in an automated system using a DAKO (DAKO, Glostrup, Denmark) autostainer in three consecutive runs per cell marker. The slides were included in random fashion in each run to avoid group-wise staining. Eosinophils, neutrophils and IL17+ cells were assessed with specific antibodies against eosinophilic peroxidase (EPX) (laboratories of NA Lee and JJ Lee, Mayo Clinic, Scottsdale, AZ), neutrophil elastase (NP57; DAKO), and IL17 (R&D Systems, Abingdon, UK). Sections were deparaffinized and, after antigen retrieval, incubated with the primary antibodies. These antibodies were detected with Envision Detection Kit (DAKO) followed by the chromogen NovaRED (Vector Labs, Burlingame, CA). EPX was detected using biotinylated antimouse IgG_1_ (Southern Biotech, Birmingham, AL) and alkaline phosphatase-labeled conjugate (DAKO) followed by permanent Red (DAKO). Sections were manually counterstained with methylgreen.

Stainings for eosinophils, neutrophils and IL17+ cells were quantified by a blinded observer using computer-assisted image analysis at a magnification of 200× (Qwin software, Leica Microsystems Imaging Solutions, Cambridge, UK or ImageScope analysis software, Aperio, Vista, CA, USA). The largest of three biopsy sections was chosen for quantification. The number of positively stained cells was counted in the submucosal area 100 μm under the basement membrane (BM) in a total area of 0.1 mm^2^ per biopsy sample. For eosinophils also numbers of EPX+ pixels were determined and expressed per total area of 0.1 mm^2^ per biopsy sample.

To identify percentages of macrophage subsets in bronchial biopsies, a general macrophage marker CD68 was used in combination with subset-specific markers as described before (6). In this previously reported study, we reported absolute numbers of double-positive cells per length of basement membrane, which generated quite some variation as some sections/patients contained many macrophages and therefore also more macrophages of specific subsets. To decrease variability between patients, for this study we reanalyzed the sections to generate relative numbers. To do this, we counted all CD68+ cells per section and all cells double-positive for CD68 and the subset-specific marker and calculated the percentage of double-positive cells of the total number of CD68+ cells present in the section. These percentages were used for further analyses. We identified Th1/Th17 inflammation-associated macrophages (formerly known as M1) using a double staining for CD68 (anti-CD68, DAKO, Heverlee, Belgium) and IRF5 (anti-IRF5, ProteinTech Europe, Manchester, UK). Th2 inflammation-associated macrophages (formerly known as M2) were identified using a double staining for CD68 (anti-CD68, Abnova, Heidelberg, Germany) and CD206 (anti-CD206, Serotec, Puchheim, Germany). Anti-inflammatory macrophages were identified using a double staining for CD68 (anti-CD68, DAKO) and IL-10 (anti-IL10, Hycult Biotech, Uden, The Netherlands) using standard immunohistochemical procedures.

In short, sections were deparaffinized and antigen retrieval was performed by overnight incubation in Tris-HCL buffer pH 9.0 at 80°C; thereafter sections were incubated with rabbit anti-IRF5 followed by mouse anti-CD68 or mouse anti-CD206 followed by rabbit anti-CD68. Next, sections were incubated with two secondary antibodies together: horseradish peroxidase (HRP)-conjugated goat-anti-mouse antibody and alkaline phosphatase (AP)-conjugated goat-anti-rabbit antibody.

For IL-10 stainings, antigen retrieval was performed by heating the sections in citrate buffer at pH 6.0 for 10 min at sub-boiling temperature. The sections were pretreated with 1% bovine serum albumin (Sigma Aldrich, Zwijndrecht, The Netherlands) and 5% milk powder in PBS for 30 min and incubated with rabbit anti-IL-10 overnight. Subsequently, the sections were incubated with mouse anti-CD68 followed by two secondary antibodies together: HRP-conjugated goat-anti-rabbit antibody and alkaline AP-conjugated goat-anti-mouse antibody.

The AP-conjugated antibodies were first visualized using an immunoalkaline phosphatase procedure with 5-bromo-4-chloro-3-indolyl-phosphate/nitro blue tetrazolium (BCIP/NBT) as chromogen (Vector, Burlingame, CA, USA). Next, the HRP-conjugated antibodies were visualized with ImmPACT NovaRED (Vector, Burlingame, CA, USA) as chromogen.

All stainings were quantified by morphometric analysis using ImageScope analysis software (Aperio, Vista, CA, USA). Two blinded observers manually counted the CD68 single-positive and subset double-positive cells in whole bronchial biopsy sections per length of intact basement membrane and extending 100 μm into the intact submucosa, excluding vessel and smooth muscle. The scores of the two observers were averaged. Data are expressed as the percentage of double-positive cells of CD68 single-positive cells.

### Statistics

To group patients, unsupervised hierarchical clustering without specifying a number of clusters was performed based on inflammatory cell counts and lung function (29, 30): Sputum eosinophils (%), biopsy eosinophils (EPX+ area), biopsy eosinophils (number of EPX+ cells), sputum neutrophils (%), biopsy neutrophils (number of NP57+ cells), baseline FEV_1_, maximum FEV_1_ from all asthma patients were used as input for the hierarchical clustering method. The data was first standardized (i.e., z-scores) and clustered by average linkage clustering and using Pearson correlation as distance metric (performed in T-MEV 4_9_0). No additional work was done to decide on an optimal number of clusters. The clusters were named using the patterns shown by z-scores, i.e., eosinophilic/mixed, paucigranulocytic, neutrophilic with low FEV1, or neutrophilic with high FEV1.

Normality of distributions was assessed using D'Agostino-Pearson omnibus test. When data were not normally distributed, log or square root transformations were performed. Asthma inflammatory subgroups were compared using a one-way ANOVA with *post-hoc* pairwise comparisons for continuous data or a Chi-square test for nominal data in ArrayStudio. Correlations were testes with a Spearman correlation test. All asthma subgroups combined and healthy individuals were compared using a Student's *t*-test. Data are shown as aligned dot plots with line at median. *P*-values < 0.05 were considered significant.

## Results

### Four Inflammatory Asthma Phenotypes Identified by Clustering

**Figure 1:**
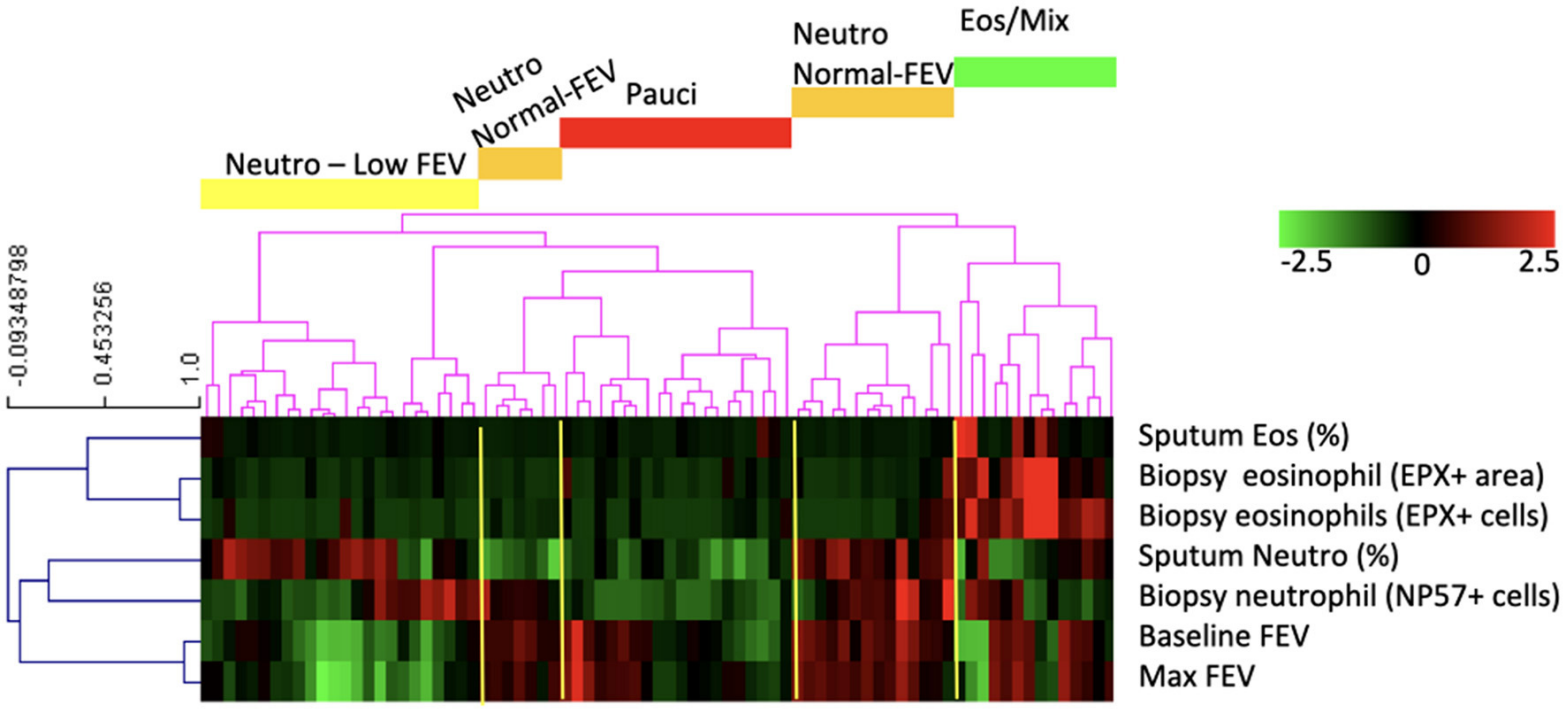
The clustering analysis based on neutrophil and eosinophil counts and lung function resulted in five groups of patients of which four had phenotypically different disease: patients with asthma characterized by mixed eosinophilic/neutrophilic inflammation (Eos/Mix), with neutrophilic asthma with low FEV_1_ (Neutro Low FEV), with neutrophilic asthma with normal FEV_1_ (Neutro Normal FEV), or with paucigranulocytic asthma (Pauci).

Our dataset consisted of 138 smoking and non-smoking patients and to identify asthma phenotypes, we used eosinophil and neutrophil cell counts from bronchial biopsies and sputum samples and pre-and post-bronchodilator FEV1. For 79 patients we had complete datasets and we clustered those patients into phenotypes. The clustering resulted in five asthma phenotypes (Figure 1): (1) asthma characterized by mixed eosinophilic and neutrophilic inflammation (Eos/Mix); (2) neutrophil-predominant asthma with low FEV_1_ (Neutro Low FEV); (3) biopsy neutrophil-predominant asthma with normal FEV_1_ (Neutro Normal FEV); (4) sputum neutrophil-predominant asthma with normal FEV_1_ (Neutro Normal FEV); (5) paucigranulocytic asthma (Pauci). We decided to combine group 3 and 4 as both had normal FEV_1_ and high numbers of neutrophils either in sputum or biopsy tissue. There is no gold standard to decide what compartment defines neutrophilic inflammation best and sputum and biopsy neutrophils did not correlate (Spearman correlation rho = 0.086, *p* = 0.44). To avoid selection bias, we therefore decided that high neutrophil counts in either sputum, tissue or both defined patients as neutrophilic.

We found no significant differences across the asthma phenotypes for inhaled corticosteroid use (ICS, as analyzed by use or by dose), atopy status, body mass index (BMI), age and sex. However, patients with neutrophilic inflammation and a low FEV_1_ had more pack years of smoking and were more often current smokers compared with the other phenotypes. This effect was not caused by inclusion of COPD patients as a diagnosis of asthma was confirmed by a pulmonologist and patients also had to have airway hyperresponsiveness. Patient characteristics of the patients with the four asthma phenotypes are described in the Table 1, together with a group of 50 healthy control subjects that have been used as a reference before (6).

### Patients With Neutrophilic Asthma and Low FEV1 Have the Most IRF5+ Macrophages and the Fewest IL10+ Macrophages of All Patients

**Figure 2:**
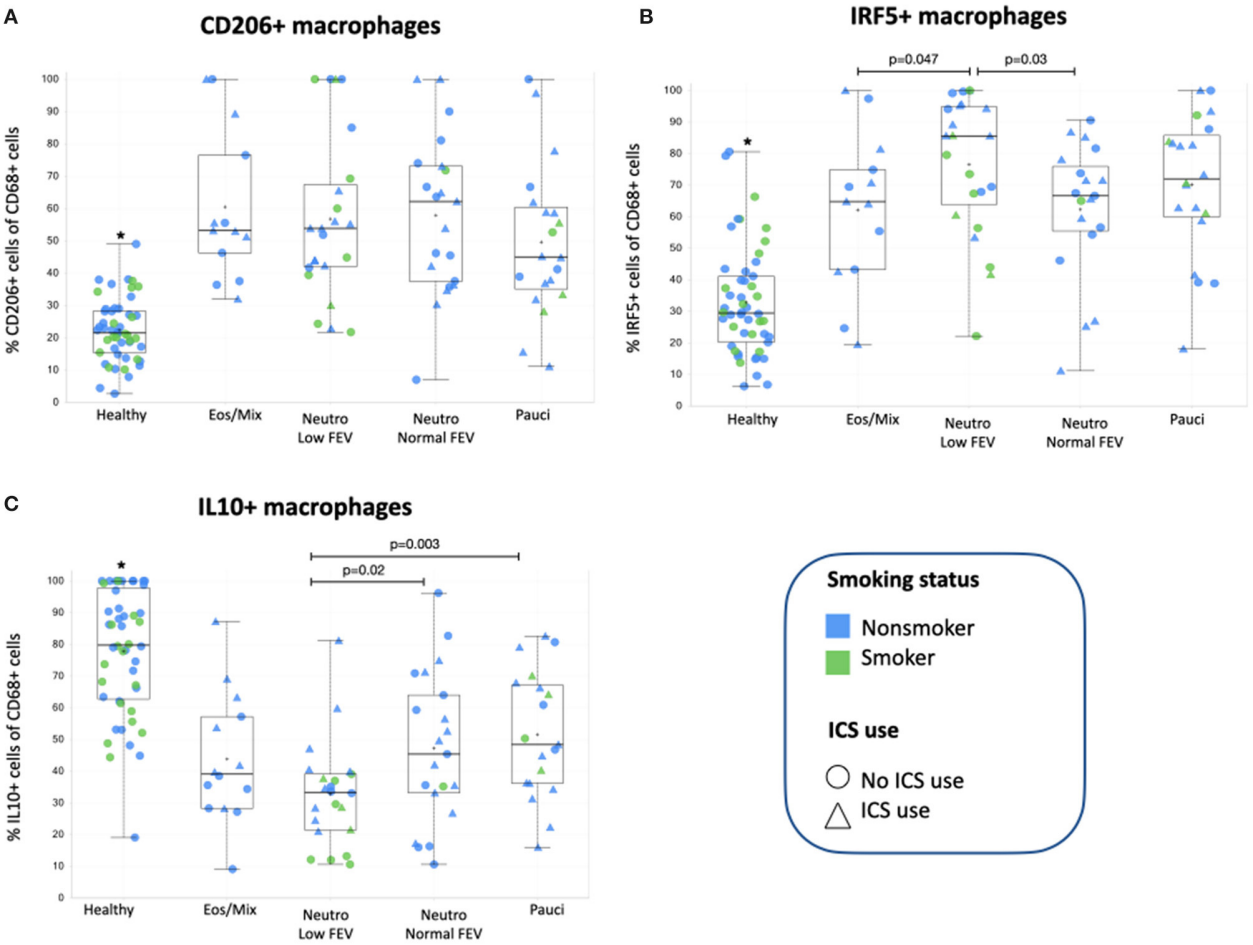
**(A)** The percentage of CD206+ macrophages present in bronchial biopsies of patients from the four subclusters and healthy controls. **(B)** The percentage of IRF5+ macrophages present in bronchial biopsies of patients from the four subclusters. **(C)** The percentage of IL10+ macrophages present in bronchial biopsies of patients from the four subclusters and healthy controls. *Healthy controls were added for comparison and are significantly different (*p* = 1.9E-17 for IRF5+, *p* = 3.9E-17 for CD206+, and *p* = 3.1E-15 for IL10+ macrophages respectively) from individuals with asthma as we have reported before (6). The asthma subgroups were compared using one-way ANOVA with *post-hoc* testing to compare groups, *p*-values < 0.05 were considered significant. ICS, inhaled corticosteroids.

We subsequently investigated how many of the different macrophage subsets were present in bronchial biopsies of these asthma patients. We first established whether the previously reported differences between asthma patients and healthy individuals were still present in this subset of 79 patients and found this to be the case (6). Asthma patients had significantly more CD206+ (*p* = 3.9E-17) and IRF5+ (*p* = 1.9E-17), and fewer IL10+ macrophages (*p* = 3.1E-15) than healthy individuals (Figure 2).

When then comparing macrophage subsets within the different asthma subgroups, we found CD206+ macrophages were not significantly different between the different patient groups (Figure 2A) and also did not correlate with the number of eosinophils in sputum or tissue (data not shown). The presence of the other two macrophage subsets was different among the asthma subgroups. We found that patients with neutrophilic asthma and low FEV1 had the most IRF5+ macrophages of all asthma subgroups, which was significantly different from patients with mixed eosinophilic asthma and patients with neutrophilic asthma and normal FEV1 (Figure 2B). This pattern was approximately reversed for IL10+ macrophages. Patients with neutrophilic asthma and low FEV1 had the fewest IL10+ macrophages of all asthma subgroups, which was significantly different from patients with paucigranulocytic asthma and patients with neutrophilic asthma and normal FEV1 (Figure 2C). Since the subgroup of patients with neutrophilic asthma and low FEV1 had significantly more current smokers and pack years than the other groups, we checked if presence of IRF5+ or IL10+ macrophages correlated with smoking history, but this was not the case. Neither did we find significant differences between smokers and never/exsmokers or patients using corticosteroids or not (data not shown).

As IL17 is associated with neutrophilic inflammation (31), we wondered whether the striking difference between the two patient groups with neutrophilic inflammation could be explained by differential presence of IL17. The number of IL17+ cells was reported on before in this patient group (32) and we studied their numbers in the 79 patients included in this dataset. However, no significant differences were found between the four subgroups of patients (Supplemental Figure 1).

## Discussion

We set out to investigate the association of different asthma phenotypes with types of macrophage activation to better understand underlying asthma endotypes. We now show that high percentages of pro-inflammatory IRF5+ macrophages and low percentages of anti-inflammatory IL10+ macrophages are significantly associated with a subgroup of asthma patients that smoke and have neutrophilic inflammation with low FEV_1_, but not with those that have neutrophilic inflammation with normal FEV_1_. These observations show that there are different types of neutrophilic asthma that can be distinguished by macrophage activation patterns suggesting differences in underlying disease mechanisms.

The association of smoking with neutrophilic asthma and lower lung function has been shown before in multiple publications, as reviewed by Polosa and Thomson (33). Remarkably this subgroup also had the highest percentage of pro-inflammatory macrophages usually associated with Th1/Th17 inflammation. We have previously shown in mice that IRF5+ macrophages are mainly present in neutrophilic inflammation (5), but we now show that just having neutrophilic inflammation is not enough as these macrophages mostly associate with smoking-related neutrophilic inflammation with low FEV_1_ and not with neutrophilic inflammation with normal FEV_1_. This suggests that smoking and/or another factor could be adding to this specific phenotype and we therefore investigated IL17 production. IL17 is an important cytokine promoting neutrophilic inflammation and plays a role in smoking-induced inflammation and has been associated with poor lung function (34, 35). However, we did not find evidence for IL17 being involved as all subgroups of asthma had similar numbers of IL17+ cells in bronchial biopsies. In addition, from the data in Figure 2 it appears non-smokers on average have higher percentages of IRF5+ macrophages than smokers within the neutrophilic low FEV1 group. Therefore, other factors seem to be responsible for the specific induction of IRF5+ macrophages in smoking-associated neutrophilic asthma. Interestingly, patients with this smoking-associated neutrophilic asthma and low FEV_1_ also had the lowest percentage of anti-inflammatory IL10+ macrophages of all subgroups. Moreover, smokers within this group seem to have the least of these macrophages as compared to non-smokers. Anti-inflammatory macrophages can be induced by corticosteroids and stimulate resolution of inflammation (7). Although patients with high neutrophils/IRF5+ macrophages and low FEV_1_ received similar ICS treatment (in dose and frequency) as the other groups, this was not associated with having better lung function or having more IL10+ macrophages. This may suggest steroid insensitivity in smoking asthmatics as has been reported before (33).

CD206+ macrophages were not significantly different between the asthma phenotypes. These macrophages are typically induced by Th2-type inflammation, characterized by high numbers of eosinophils and levels of cytokines like IL4 and IL13, but we did not find more CD206+ macrophages in bronchial biopsies of patients with eosinophilic/mixed inflammation. This may suggest that these patients do not have dominant Th2-inflammation as compared to the other phenotypes, possibly as a consequence of ICS use and being relatively well-controlled. An alternative explanation is that our marker CD206 (the mannose receptor) is less efficient in picking up differences in higher levels of Th2 inflammation. The fact that all asthma subgroups had significantly more CD206+ macrophages than healthy controls does indicate that the inflammation in all asthma subgroups has some level of Th2 inflammation.

A limitation of our study is the somewhat artificial subdivision of macrophages into three activation states while *in vivo* they appear as a continuous spectrum rather than discrete subsets (36). This is also evident from the fact that the sum of the three subsets often adds up to over 100%, suggesting overlap in markers between subsets. A way to move the field forward would be the use of dozens of markers with tissue cytof (37) and subsequent unbiased clustering of macrophage populations based on expression of these dozens of markers. This combines the advantages of flow cytometry without the risk of losing specific cells during isolation and with the preservation of spatial information.

In conclusion, our study has shown that neutrophilic asthma with low FEV1 is associated with high numbers of IRF5+, and low numbers of IL10+ macrophages, which may be the result of the combined effects of smoking and having asthma.

## Data Availability Statement

Anonymized individual participant data from this study can be requested for further research from the corresponding author.

## Ethics Statement

This study involving human participants was reviewed and approved by Medical Ethical Committee of the UMCG (METc 2007/007, clinicaltrials.gov NCT00848406). The patients/participants provided their written informed consent to participate in this study.

## Author Contributions

BM, MB, NT, and AO: experimental design. NH and MB: collection of patient material and data. WT and FF: processing of patient material. CD, TV, and FF: experimental work. NT and BM: analyses of data and manuscript writing. All authors revised the manuscript critically for important intellectual content and approved the final version of the manuscript.

## Conflict of Interest

AO and NT were employed by the company GlaxoSmithKline at the time of data analysis and manuscript writing. The remaining authors declare that the research was conducted in the absence of any commercial or financial relationships that could be construed as a potential conflict of interest.

## References

[1] Fitzpatrick A Moore W. Severe Asthma Phenotypes - How Should They Guide Evaluation and Treatment. J Allergy Clin Immunol Pract. (2017) 5:901–8. 10.1016/j.jaip.2017.05.015 28689840 PMC5541906

[2] Shaw D Sousa A Fowler S Fleming L Roberts G Corfield J. Clinical and inflammatory characteristics of the European U-BIOPRED adult severe asthma cohort. Eur Respir J. (2015) 46:1308–21. 10.1183/13993003.00779-2015 26357963

[3] Lötvall J Akdis C Bacharier L Bjermer L Casale T Custovic A. Asthma endotypes: a new approach to classification of disease entities within the asthma syndrome. J Allergy Clin Immunol. (2011) 127:355–60. 10.1016/j.jaci.2010.11.037 21281866

[4] Tang HHF Sly PD Holt PG Holt KE Inouye M. Systems biology and big data in asthma and allergy: recent discoveries and emerging challenges. Eur Respir J. (2020) 55:1900844. 10.1183/13993003.00844-2019 31619470

[5] Draijer C Robbe P Boorsma CE Hylkema MN Melgert BN. Dual role of YM1+ M2 macrophages in allergic lung inflammation. Sci Rep. (2018) 8:5105. 10.1038/s41598-018-23269-7 29572536 PMC5865212

[6] Draijer C Boorsma C Robbe P Timens W Hylkema M ten Hacken N. Human asthma is characterized by more IRF5+ M1 and CD206+ M2 macrophages and less IL-10+ M2-like macrophages around airways compared with healthy airways. J Allergy Clin Immunol. (2017) 140:280-3.e3. 10.1016/j.jaci.2016.11.020 28007476

[7] Draijer C Boorsma C Reker-Smit C Post E Poelstra K Melgert B. PGE2-treated macrophages inhibit development of allergic lung inflammation in mice. J Leukoc Biol. (2016) 100:95–102. 10.1189/jlb.3MAB1115-505R 26931576 PMC6608083

[8] Draijer C Robbe P Boorsma CE Hylkema MN Melgert BN. Characterization of macrophage phenotypes in three murine models of house-dust-mite-induced asthma. Mediators Inflamm. (2013) 2013:632049. 10.1155/2013/632049 23533309 PMC3600196

[9] Melgert B ten Hacken N Rutgers B Timens W Postma D Hylkema M. More alternative activation of macrophages in lungs of asthmatic patients. J Allergy Clin Immunol. (2011) 127:831–3. 10.1016/j.jaci.2010.10.045 21167569

[10] Melgert B Oriss T Qi Z Dixon-McCarthy B Geerlings M Hylkema M. Macrophages: regulators of sex differences in asthma? Am J Respir Cell Mol Biol. (2010) 42:595–603. 10.1165/rcmb.2009-0016OC 19574533 PMC2874445

[11] Braza F Dirou S Forest V Sauzeau V Hassoun D Chesné J. Mesenchymal stem cells induce suppressive macrophages through phagocytosis in a mouse model of asthma. Stem Cells. (2016) 34:1836–45. 10.1002/stem.2344 26891455

[12] Zdrenghea M Makrinioti H Muresan A Johnston S Stanciu L. The role of macrophage IL-10/innate IFN interplay during virus-induced asthma. Rev Med Virol. (2015) 25:33–49. 10.1002/rmv.1817 25430775 PMC4316183

[13] Robbe P Draijer C Borg T Luinge M Timens W Wouters I. Distinct macrophage phenotypes in allergic and nonallergic lung inflammation. Am J Physiol Lung Cell Mol Physiol. (2015) 308:L358–67. 10.1152/ajplung.00341.2014 25502502

[14] Zaslona Z Przybranowski S Wilke C van Rooijen N Teitz-Tennenbaum S Osterholzer J. Resident alveolar macrophages suppress, whereas recruited monocytes promote, allergic lung inflammation in murine models of asthma. J Immunol. (2014) 193:4245–53. 10.4049/jimmunol.1400580 25225663 PMC4185233

[15] Bedoret D Wallemacq H Marichal T Desmet C Quesada Calvo F Henry E. Lung interstitial macrophages alter dendritic cell functions to prevent airway allergy in mice. J Clin Investig. (2009) 119:3723–38. 10.1172/JCI39717 19907079 PMC2786798

[16] Goleva E Hauk P Hall C Liu A Riches D Martin R. Corticosteroid-resistant asthma is associated with classical antimicrobial activation of airway macrophages. J Allergy Clin Immunol. (2008) 122:550-9.e3. 10.1016/j.jaci.2008.07.007 18774390 PMC3930345

[17] Korf JE Pynaert G Tournoy K Boonefaes T Van Oosterhout A Ginneberge D. Macrophage reprogramming by mycolic acid promotes a tolerogenic response in experimental asthma. Am J Respir Crit Care Med. (2006) 174:152–60. 10.1164/rccm.200507-1175OC 16675779

[18] Vissers J van Esch B Hofman G van Oosterhout A. Macrophages induce an allergen-specific and long-term suppression in a mouse asthma model. Eur Resp J. (2005) 26:1040–6. 10.1183/09031936.05.00089304 16319333

[19] Careau E Bissonnette EY. Adoptive transfer of alveolar macrophages abrogates bronchial hyperresponsiveness. Am J Respir Cell Mol Biol. (2004) 31:22–7. 10.1165/rcmb.2003-0229OC 14962974

[20] Moreira AP Cavassani KA Hullinger R Rosada RS Fong DJ Murray L. Serum amyloid P attenuates M2 macrophage activation and protects against fungal spore-induced allergic airway disease. J Allergy Clin Immunol. (2010) 126:712–21.e7. 10.1016/j.jaci.2010.06.010 20673988

[21] Staples KJ Hinks TS Ward JA Gunn V Smith C Djukanović R. Phenotypic characterization of lung macrophages in asthmatic patients: overexpression of CCL17. J Allergy Clin Immunol. (2012) 130:1404–12.e7. 10.1016/j.jaci.2012.07.023 22981793 PMC3805016

[22] Boonpiyathad T Sözener ZC Satitsuksanoa P Akdis CA. Immunologic mechanisms in asthma. Semin Immunol. (2019) 46:101333. 10.1016/j.smim.2019.101333 31703832

[23] Broekema M Timens W Vonk J Volbeda F Lodewijk M Hylkema M. Persisting remodeling and less airway wall eosinophil activation in complete remission of asthma. Am J Respir Crit Care Med. (2011) 183:310–6. 10.1164/rccm.201003-0494OC 20813885

[24] Broekema M Volbeda F Timens W Dijkstra A Lee N Lee J. Airway eosinophilia in remission and progression of asthma: accumulation with a fast decline of FEV(1). Respir Med. (2010) 104:1254–62. 10.1016/j.rmed.2010.03.030 20434897

[25] Broekema M ten Hacken N Volbeda F Lodewijk M Hylkema M Postma D. Airway epithelial changes in smokers but not in ex-smokers with asthma. Am J Respir Crit Care Med. (2009) 180:1170–8. 10.1164/rccm.200906-0828OC 19797761

[26] Meijer R Kerstjens H Arends L Kauffman H Koëter G Postma D. Effects of inhaled fluticasone and oral prednisolone on clinical and inflammatory parameters in patients with asthma. Thorax. (1999) 54:894–9. 10.1136/thx.54.10.894 10491451 PMC1745367

[27] Quanjer P Tammeling G Cotes J Pedersen O Peslin R Yernault J. Lung volumes and forced ventilatory flows. Work Group on Standardization of Respiratory Function Tests. European Community for Coal and Steel. Official position of the European Respiratory Society. Rev Mal Respir. (1994) 11(Suppl. 3):5–40. 10.1183/09041950.005s1693 7973051

[28] Grol M Gerritsen J Vonk J Schouten J Koëter G Rijcken B. Risk factors for growth and decline of lung function in asthmatic individuals up to age 42 years. A 30-year follow-up study. Am J Respir Crit Care Med. (1999) 160:1830–7. 10.1164/ajrccm.160.6.9812100 10588593

[29] Saeed AI Sharov V White J Li J Liang W Bhagabati N. TM4: a free, open-source system for microarray data management and analysis. Biotechniques. (2003) 34:374–8. 10.2144/03342mt01 12613259

[30] Eisen MB Spellman PT Brown PO Botstein D. Cluster analysis and display of genome-wide expression patterns. Proc Natl Acad Sci USA. (1998) 95:14863–8. 10.1073/pnas.95.25.14863 9843981 PMC24541

[31] Gurczynski SJ Moore BB. IL-17 in the lung: the good, the bad, and the ugly. Am J Physiol Lung Cell Mol Physiol. (2018) 314:L6–16. 10.1152/ajplung.00344.2017 28860146 PMC6048455

[32] Fattahi F Brandsma C Lodewijk M Reinders-Luinge M Postma D Timens W. Atopy and inhaled corticosteroid use associate with fewer IL-17+ cells in asthmatic airways. PLoS ONE. (2016) 11:e0161433. 10.1371/journal.pone.0161433 27552197 PMC4994949

[33] Polosa R Thomson N. Smoking and asthma: dangerous liaisons. Eur Respir J. (2013) 41:716–26. 10.1183/09031936.00073312 22903959

[34] Sorbello V Ciprandi G Di Stefano A Massaglia G Favatà G Conticello S. Nasal IL-17F is related to bronchial IL-17F/neutrophilia and exacerbations in stable atopic severe asthma. Allergy. (2015) 70:236–40. 10.1111/all.12547 25394579

[35] Shan M Yuan X Song L Roberts L Zarinkamar N Seryshev A. Cigarette smoke induction of osteopontin (SPP1) mediates T(H)17 inflammation in human and experimental emphysema. Sci Transl Med. (2012) 4:117ra9. 10.1126/scitranslmed.3003041 22261033 PMC3956594

[36] Murray P Allen J Biswas S Fisher E Gilroy D Goerdt S. Macrophage activation and polarization: nomenclature and experimental guidelines. Immunity. (2014) 41:14–20. 10.1016/j.immuni.2014.06.008 25035950 PMC4123412

[37] Giesen C Wang HA Schapiro D Zivanovic N Jacobs A Hattendorf B. Highly multiplexed imaging of tumor tissues with subcellular resolution by mass cytometry. Nat Methods. (2014) 11:417–22. 10.1038/nmeth.2869 24584193

