# Supplementary material for: Neutrophilic Asthma Is Associated With Smoking, High Numbers of IRF5+, and Low Numbers of IL10+ Macrophages
Source: Front Allergy. 2021 Jun 21;2:676930. doi: 10.3389/falgy.2021.676930 (PMC8974785; doi:10.3389/falgy.2021.676930)
Supplement: Supplementary file 1 [file Data_Sheet_1.PDF]

Supplemental Material

Neutrophilic asthma is associated with smoking, high numbers of IRF5+, and low numbers of IL10+ macrophages

Nil Turan[1], T. Anienke van der Veen[2,5], Christina Draijer[2,5], Fatemeh Fattahi[3,4,5], Nick H. Ten Hacken[3,5], Wim Timens[4,5], Antoon J. van Oosterhout[1], Maarten van den Berge[3,5], Barbro N. Melgert[2,4,5*]

[1]GlaxoSmithKline, Allergic Inflammation Discovery Performance Unit, Respiratory Therapy Area, Gunnels Wood Road, Stevenage, Hertfordshire, SG1 2NY, UK

[2]University of Groningen, Groningen Research Institute for Pharmacy, Department of Molecular Pharmacology, Antonius Deusinglaan 1, 9713 AV Groningen, The Netherlands

[3]University of Groningen, University Medical Center Groningen, Department of Pulmonology, Hanzeplein 1, 9713 GZ Groningen, The Netherlands

[4]University of Groningen, University Medical Center Groningen, Department of Pathology and Medical Biology, Hanzeplein 1, 9713 GZ Groningen, The Netherlands

[5]University of Groningen, University Medical Center Groningen, Groningen Research Institute for Asthma and COPD (GRIAC), Hanzeplein 1, 9713 GZ Groningen, The Netherlands

*** Correspondence:**
Prof. Dr. B.N. Melgert
Department of Molecular Pharmacology
University of Groningen
Antonius Deusinglaan 1
9713 AV Groningen
The Netherlands
Tel: +31-50-3632947
Fax: +31-50-3633247
Email: b.n.melgert@rug.nl

Keywords: Biopsy; Asthma; Inflammatory endotypes; Macrophages subtypes; Neutrophils; FEV_1

Supplemental material

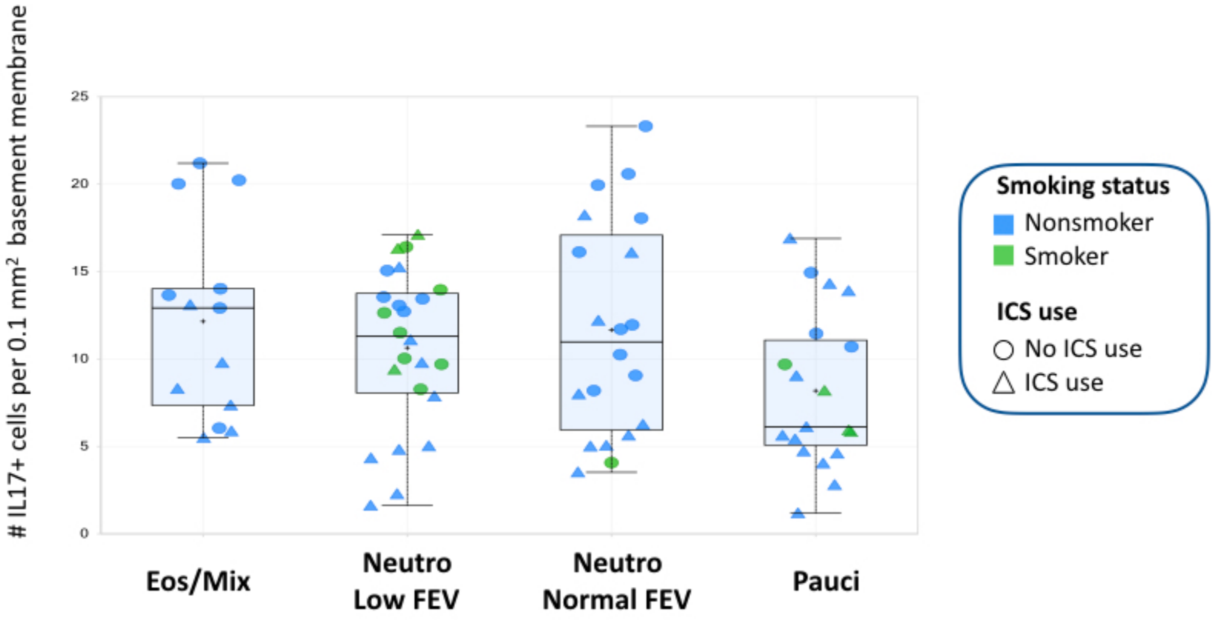

Figure S1: *The number IL17+ cells present in bronchial biopsies of patients from the four asthma subclusters.*
